# Coronavirus Disease 2019 (COVID-19) Diagnostic Tools: A Focus on Detection Technologies and Limitations

**DOI:** 10.3390/cimb43020053

**Published:** 2021-07-20

**Authors:** Ahmad Qasem, Ameera M. Shaw, Erij Elkamel, Saleh A. Naser

**Affiliations:** Division of Molecular Microbiology, Burnett School of Biomedical Sciences, College of Medicine, University of Central Florida, 4110 Libra Drive, Orlando, FL 32816, USA; ahmadqasem@knights.ucf.edu (A.Q.); amshaw@knights.ucf.edu (A.M.S.); erijelkamel@knights.ucf.edu (E.E.)

**Keywords:** COVID-19, SARS-CoV-2, pandemics, diagnostic tools, PCR, LAMP, FICA, mNGS

## Abstract

The ongoing coronavirus disease (COVID-19) pandemic caused by severe acute respiratory syndrome coronavirus 2 (SARS-CoV-2) poses a severe threat to human health and the global economy and has resulted in overwhelming stress on health care systems worldwide. Despite the global health catastrophe, especially in the number of infections and fatalities, the COVID-19 pandemic has also revolutionized research and discovery with remarkable success in diagnostics, treatments, and vaccine development. The use of many diagnostic methods has helped establish public health guidelines to mitigate the spread of COVID-19. However, limited information has been shared about these methods, and there is a need for the scientific community to learn about these technologies, in addition to their sensitivity, specificity, and limitations. This review article is focused on providing insights into the major methods used for SARS-CoV-2 detection. We describe in detail the core principle of each method, including molecular and serological approaches, along with reported claims about the rates of false negatives and false positives, the types of specimens needed, and the level of technology and the time required to perform each test. Although this study will not rank or prioritize these methods, the information will help in the development of guidelines and diagnostic protocols in clinical settings and reference laboratories.

## 1. Introduction

In December 2019, a group of patients in Hubei Province, China, presented with cough, fever, and shortness of breath [1]. Computed tomography (CT) scans revealed profuse and confluent pulmonary abnormalities, which initially led to the diagnosis of bacterial pneumonia [2]. However, common suspected etiological agents, such as *Haemophilus influenzae*, *Streptococcus pneumoniae*, and adenoviruses, were not detected in bacterial culture tests or viral nucleic acid analyses [1]. Therefore, the cause of this pneumonia was of unknown origin until bronchoalveolar lavage fluid (BALF) samples were analyzed, showing a new pathogen that had an almost identical genetic sequence to betacoronavirus (β-CoV) B lineage [3]. This newly emerging virus shares 80% genomic similarity with the severe acute respiratory syndrome virus (SARS-CoV), 50% with the Middle East respiratory syndrome coronavirus (MERS-CoV), and 96% with the bat coronavirus RaTG13 [1,3]. Further sequencing analysis revealed that the virus belongs to a family of viruses known as Coronaviridae, which was later identified in February 2020 as the severe acute respiratory syndrome coronavirus 2 (SARS-CoV-2) by the International Virus Classification Commission [4]. The disease caused by SARS-CoV-2 is known as coronavirus disease 2019 (COVID-19) and has since become a global pandemic, infecting over 188 million people and resulting in at least 4.05 million deaths worldwide [5].

**Figure 1:**
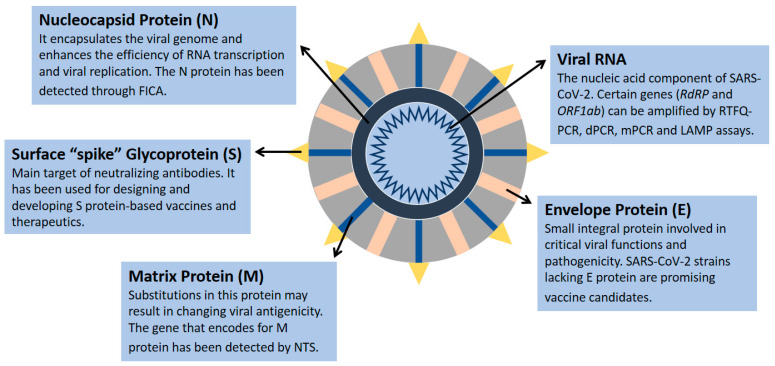
SARS-CoV-2 structural proteins and genomic component. Data were obtained from several published reports [1,6,7,8,9,10].

The nucleic acid component of SARS-CoV-2 is composed of nearly 30,000 nucleotides forming a single-stranded positive-sense RNA, which encodes 27 proteins, including 4 structural proteins (nucleocapsid protein (N), matrix protein (M), small envelope protein (E), and surface glycoprotein (S)) (Figure 1) and RNA-dependent RNA polymerase (RdRP) [1,6,7,8,9,10]. The genes encoding the E, M, and N proteins are generally conserved and only involved in regular viral functions, whereas the S gene is more divergent with less than 75% sequence similarity compared with other coronaviruses [1,6]. The receptor-binding domain of the S spike protein mediates the viral attachment of SARS-CoV-2 to human angiotensin-converting enzyme 2 (ACE2) receptors, and it is a major target of neutralizing antibodies [11]. Several SARS-CoV-2 strains have been sequenced, revealing 99.9% homology among viral strains [12]. However, diverse viral genomic changes have started to emerge, resulting in new variant strains, such as the Zeta variant (B.1.1.28) and the Delta variant (B.1.617.2), which may be more infectious and deadly than the first identified viral strain [12,13].

Expanding COVID-19 diagnostic capacity is a crucial priority, and understanding the difference between detection results of SARS-CoV-2 infection is imperative for health care professionals to perform accurate interpretations of viral exposure and immunological responses, which may ultimately influence the selection of appropriate treatment options. Since this is a rapidly developing topic, the current findings may be useful for implementing certain strategies to limit the rapid spread of COVID-19. In this review article, we summarize the clinical diagnostic advances and detection technologies of SARS-CoV-2 infection based on four main categories: nucleic acid amplification technologies, immunological detection techniques, sequencing detection methods, and additional alternative methods. In accordance with recent reports, we briefly discuss the strengths and weaknesses of these major diagnostic applications that play a significant role in identifying SARS-CoV-2 infection, therefore helping to counter the spread of COVID-19.

## 2. Nucleic Acid Amplification Technologies

### 2.1. Polymerase Chain Reaction (PCR) Methods

#### 2.1.1. Fluorescence-Based Real-Time Quantitative PCR (qPCR)

Quantitative real-time PCR (qPCR) amplifies a target nucleic acid fragment while quantifying it in real time [14]. This quantitation can either be relative, analyzing the amount of the target compared with a reference sample, or absolute, determining the exact amount of the target with an unknown concentration in relation to a control nucleic acid with a known concentration [14]. Absolute quantitation allows for monitoring the progression of infection by expressing the viral load in units familiar to scientists and clinicians, therefore allowing for a distinction to be made between active and persistent infections [14].

In 2003, two fluorescence-based qPCR assays were developed to measure the SARS-CoV RNA concentration in plasma and serum samples of SARS patients [15]. The assays targeted regions of the viral genome that included polymerase or nucleocapsid genes [15]. Upon extraction of viral RNA from patient samples, SARS-CoV RNA was successfully detected by both qPCR systems (78% by the polymerase system and 87% by the nucleocapsid system) with higher detection rates in serum samples compared with plasma samples [15]. The qPCR was also found to be more sensitive in detecting SARS-CoV in RNA isolates of nasopharyngeal aspirates and stool samples of SARS patients compared with reverse transcription PCR (RT-PCR) [16]. Moreover, testing more than one respiratory specimen maximized the sensitivity of RT-PCR assays used for SARS-CoV detection [17,18].

The presence of SARS-CoV-2 was successfully detected using fluorescence-based qPCR assays targeting either the RdRP or E genes [19]. Synthetically derived SARS-COV-2 RNA standards were used to determine the limit of detection, which was similar in both gene assays with 3.6 copies for the RdRP gene and 3.9 for the E gene, showing high sensitivity for both qPCR assays [19]. The RdRP gene assay was also formulated with a SARS-CoV-2-specific probe that effectively discriminated between SARS-CoV and SARS-CoV-2 such that it only detected SARS-CoV-2 RNA transcripts [19]. Additionally, researchers found exclusivity of SARS-CoV-2, with no false positives, when using the SARS-CoV-2 RdRP and E gene qPCR assays to test for cross-reactivity in clinical samples with a broad range of known respiratory agents (viral and bacterial) [19]. RNA was isolated from sputum, nose, and throat swabs of individuals in various countries and prepared cell culture supernatants containing all endemic human coronaviruses (HCoV-HKU1, HCoV-OC43, HCoV-NL63, HCoV-229E, and MERS-CoV) [19].

On 4 February 2020, the Food and Drug Administration (FDA) issued emergency use authorization for the Center for Disease Control (CDC) SARS-CoV-2 Real-Time Quantitative Reverse Transcription PCR (RT-qPCR) Diagnostic Panel to be used in non-CDC laboratories as a coronavirus diagnostic [20]. RNA isolated from respiratory samples (nasopharyngeal or oropharyngeal swabs, sputum, lower respiratory aspirates, bronchoalveolar lavage, and nasopharyngeal or nasal aspirates) is reverse-transcribed into cDNA and then amplified via qPCR [21]. This method utilizes oligonucleotide primers and probes targeted to regions of the viral N gene [21]. Upon annealing of the probe to its target sequence, Taq polymerase’s 5′ nuclease activity degrades the probe, thereby causing separation of the reporter dye from the quencher dye, resulting in a fluorescent signal [21]. Fluorescence intensity increases proportionally with the cleavage of reporter dyes from their probes in each amplification cycle in response to the increased concentration of the amplicon [19]. Previous studies have illustrated the efficacy of targeting the ORF1b gene for early disease detection [16]. Additionally, the ORF1b gene has been found to have considerable stability, which is extremely advantageous for laboratory testing [16]. Quantifying both the RdRP and E genes has produced accurate and equitable results [19]. However, quantifying the N gene has been deemed inauspicious for clinical diagnosis because of its decreased sensitivity [19].

**Figure 2:**
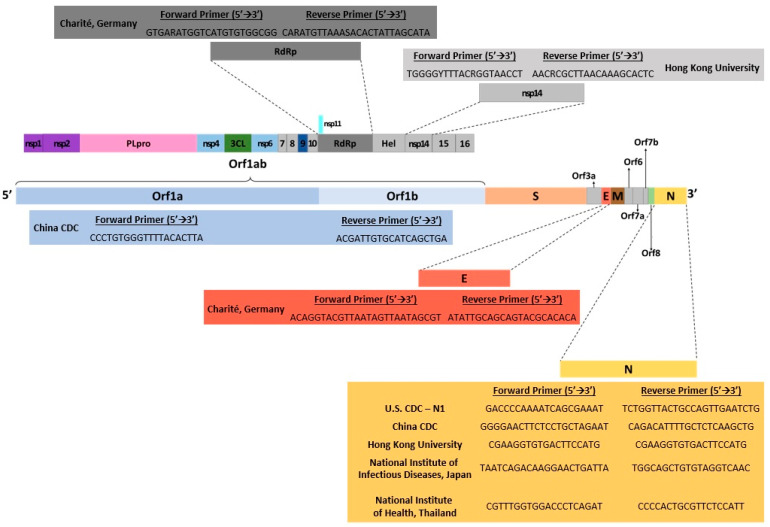
Illustration of SARS-CoV-2 genomic map with gene targets of diagnostic RT-PCR tests and their corresponding 5′→3′ forward/reverse primers. Data were obtained from several published reports [6,19,20,21,22,24].

Some of the drawbacks of RT-qPCR include having false-positive or negative results that can occur if there is contamination of a specimen due to improper collection [21]. High disease prevalence can also increase the likelihood of false negatives, while a moderate to low prevalence can increase the rate of false positives [22]. The initial start-up expense for RT-qPCR could also be problematic for smaller, low-throughput laboratories [14]. Using one-tube RT-qPCR protocols has been found to minimize environmental contamination, which increases test sensitivity [23]. Ultimately, RT-qPCR has become widely accepted as a gold standard for nucleic acid detection from various sources due to its accuracy, sensitivity, and decreased risk of contamination [14]. Several RT-qPCR kits have been designed to detect SARS-CoV-2 genetically and have also been used in several countries worldwide (Figure 2).

#### 2.1.2. Digital PCR (dPCR)

Digital polymerase chain reaction (dPCR) enables absolute quantification of nucleic acids without the need for standard curves or relative threshold (Ct) values, therefore overcoming some of the limitations of RT-qPCR [25,26,27]. This method, referred to as chamber-based digital PCR (cdPCR), involves isolating nucleic acid molecules using a limiting dilution technique to physically partition samples on a microfluidic chip so that most reactions contain one or zero molecules [25,27,28,29]. Alternatively, nucleic acid samples can be randomly mixed into a water-in-oil emulsion to partition molecules into thousands of nanodroplets in a technique called droplet digital PCR (ddPCR) [30]. PCR is then performed to endpoint in all compartments/droplets, and then fluorescence measurement is used to determine the number of amplification-positive and negative signals [30,31,32]. Poisson distribution statistical analysis is employed to calculate the absolute quantification of nucleic acid molecules [24,31].

The applications of dPCR include quantitation of gene copy number variation, gene expression, RNA/microRNA quantitation, and rare sequence detection such as viral load analysis from clinical samples [33]. Therefore, dPCR has been used as a diagnostic method for SARS-CoV-2 and may arguably serve as a superior technique to RT-qPCR, which is currently the gold standard for the detection of SARS-CoV-2 according to the World Health Organization (WHO) and the CDC [34,35,36,37,38].

Reverse transcription dPCR (RT-dPCR) was compared with RT-qPCR for the detection of the ORF1ab sequence, N gene, and E gene of the SARS-CoV-2 genome from 194 clinical samples [34]. RT-dPCR exhibited higher overall diagnostic accuracy and sensitivity (93% and 90%, respectively) [34]. Additionally, the limit of detection (LoD) for RT-dPCR was reported to be 2 copies/reaction for all genes tested [34]. Another study by Suo et al. using ddPCR also concluded that this technique has several advantages over RT-qPCR for the clinical diagnosis of SARS-CoV-2 [35]. The LoD of ddPCR was significantly higher for ORF1ab and N genes (2.1 and 1.8 copies/reaction, respectively) compared with the LoD of RT-qPCR (1039 and 873.2 copies/reaction, respectively). Based on 77 clinical samples, ddPCR was about 500 times more sensitive than RT-qPCR for SARS-CoV-2 detection [35]. Additionally, the negative predictive value (NPV) of ddPCR was higher than that of RT-qPCR, indicating that ddPCR has a lower false-negative rate and would reduce the risk of potential viral transmission if used for clinical diagnosis [35].

Due to its higher sensitivity and accuracy, dPCR is suitable for detecting low viral loads, therefore allowing for early treatment and reduced risk of community transmission [34,35,36]. This contrasts with RT-qPCR, which has a higher rate of false-negative diagnostic results [39]. The dPCR is also advantageous as it does not require a calibration standard and generates absolute quantifications through Poisson distribution, therefore allowing for comparisons to be made between results produced from different laboratories or from different dates, which is not the case for Ct values produced by RT-qPCR [40]. However, dPCR relies on more expensive instrumentation and reagents and uses a more complex method that requires multiple steps, ultimately slowing the workflow and increasing the time needed to produce results [41].

#### 2.1.3. Multiplex PCR (mPCR)

Multiplex PCR (mPCR) is used to amplify more than one target sequence using two or more primer sets in a single reaction. This method was first employed to detect deletions in the human dystrophin gene [42] and has since become a firmly established technique. By simultaneously amplifying several target genes in the same reaction, mPCR can produce savings in time, effort, and cost in the laboratory. It also has very high sensitivity and specificity, which further reinforces the effectiveness and practicality of this approach [43,44,45,46]. Applications of mPCR include nucleic acid diagnostics, such as mutation and polymorphism analysis and RNA detection [45,47,48,49]. It has also been used for the diagnosis of infectious diseases, such as through the identification of viruses, bacteria, and parasites [50,51].

Multiplex assays have recently been designed to identify multiple gene targets for the detection of SARS-CoV-2 RNA in clinical samples. Soon after the emergence of the COVID-19 epidemic, the ARTIC Network proposed 98 multiplexed PCR primer pairs for whole-genome analysis of SARS-CoV-2 [52]. Although the proposed primer set is effective for viral detection in clinical samples containing relatively high viral loads, PCR products 18 and 76, which code for nonstructural protein 3 (nsp3) in ORF1a and the S protein, have exhibited reductions in amplification efficiencies due to heterodimer formation because of a 10 bp overlap between these primers [53]. Itokawa et al. recommended replacing one of these primers with a newly designed primer and demonstrated that this resulted in improved coverage at both regions targeted by these PCR products [53]. Another study by Tyson et al. proposed further enhancements to the ARTIC mPCR method involving a modified primer scheme with 22 additional primers for increased genome coverage, streamlined workflow, and strategies to lower costs and improve scalability, ultimately improving accuracy and efficiency [54].

Another mPCR assay was proposed by Li et al. using 343 primer pairs that were shown to be efficient at detecting SARS-CoV-2 at low copy numbers [55]. Their method demonstrated high coverage and specificity of the S and N genes, with medians of 99% and 99.8%, respectively, and improved sensitivity compared with RT-qPCR [55]. RT-qPCR exhibits a positive rate of only 47–59% due to the presence of false-negative results, therefore requiring repeated testing of samples [56,57,58]. Due to the use of multiple primer pairs in the mPCR strategy, it is unlikely that false-negative results will be produced from clinical samples containing low viral loads [54,55,59]. Unlike RT-qPCR, mPCR can effectively detect SARS-CoV-2 even in the presence of mutations in primer binding sites and may even detect degraded viral genomes [55]. The use of a triplex RT-qPCR assay targeting viral nucleocapsid genes, N1 and N2, showed 98.4% accuracy and improved assay throughput compared with a singleplex RT-qPCR assay using just one primer in [59]. Another study designed four primer sets targeting the essential genes of SARS-CoV-2, RdRP, S, N, and E for accurate and efficient viral detection via an mPCR-based protocol [60]. With the emergence of more transmissible and infectious SARS-CoV-2 variants that are able to escape the immune response, there is an urgent need for targeted detection of circulating lineages, which can be achieved through mPCR assays.

Overall, the use of mPCR for SARS-CoV-2 detection is associated with reduced reagent consumption, cost-efficiency, simple workflow, high sensitivity, diagnostic accuracy, and high throughput [54,55,59]. However, there is some loss in genome coverage, the potential for contamination due to the method in which PCR plates must be prepared, and the risk of competition between reaction components may affect the amplification process and result in reaction failure [54,60]. The specificity of mPCR assays can be improved by selecting multiple targets, or by identifying new genomic regions, such as nonstructural protein 2 (nsp2), which led to the development of the COVID-19-nsp2 assay [61]. Optimizing primer and reaction conditions for effective mPCR can also be time-consuming and resource-intensive [43], but this can be overcome by employing the methodology of a previously published study for SARS-CoV-2 detection.

### 2.2. Loop-Mediated Isothermal Amplification (LAMP)

To overcome conventional PCR diagnostic limitations, a quicker and more cost-effective method has been utilized to test SARS-CoV-2 infection without an extensive sample processing or the need for highly skilled personnel. Loop-mediated isothermal amplification (LAMP) is a nucleic acid amplification technology that is carried out in isothermal conditions and does not require changes in cycle temperatures [62]. This method can be performed in a single step that involves incubating the nucleic acid sample, amplification primers, and DNA polymerase in one test tube at an optimal LAMP temperature, which is usually around 65 °C [63]. By employing this technique, it eliminates the need for specific thermal cycler equipment or a narrow sample pH range [64]. Moreover, this flexible method provides similar sensitivity and specificity levels to that of the RT-qPCR assay [65].

A new LAMP test was developed for COVID-19 detection that can be performed within 30 min [66]. Specific LAMP primers were designed to broadly target SARS-CoV-2 based on the sequence of 23 SARS-CoV-2 strains obtained from GenBank [66]. Healthy human samples spiked with an oligonucleotide of GenBank MN908947.3, which was used as a positive control for COVID-19, while simulated negative control samples were prepared using other coronaviruses, including MERS and murine coronavirus (MHV). This method is known as RT-LAMP and demonstrates high specificity towards SARS-CoV-2 as validated by both fluorescence and gel electrophoresis [66]. The assay also exhibited very high sensitivity and identified SARS-CoV-2 infection in various spiked sample types, including NP swab, saliva, urine, and serum samples [64]. Similar LAMP assays were recently developed, including an isothermal LAMP-based method for COVID-19 (iLACO assay) and a one-pot RT-LAMP assay [67,68].

**Figure 3:**
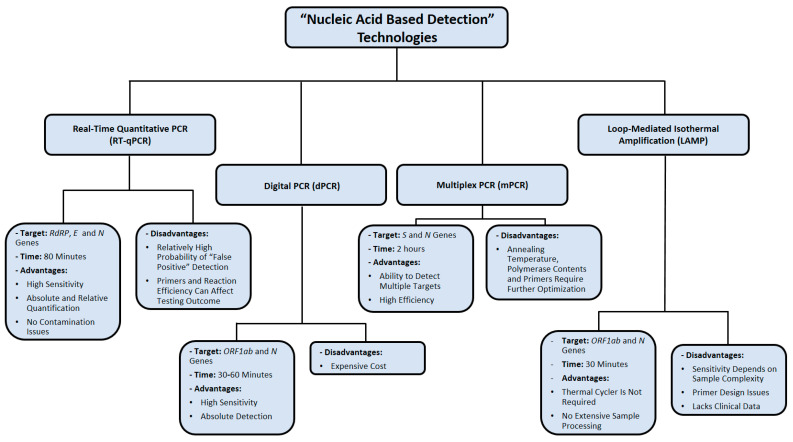
Summary of nucleic acid amplification technologies used in diagnosing SARS-CoV-2 infection. Data were obtained from several published reports [17,18,19,22,34,35,36,37,52,53,54,55,66,67,68,69].

The major limitation of LAMP assays is the difficulty of preparing appropriate primer designs, therefore reducing its reproducibility in multiplexing assays [69]. Additionally, LAMP seems to be less sensitive than PCR when the sample being tested is more complex, such as blood or tissue samples [69]. Up to this point, several LAMP-based assays are validated using computational resources only, without implementing any clinical data for final performance analysis [65]. The nucleic-acid-based detection methods used for SARS-CoV-2 detection are summarized in Figure 3.

## 3. Immunological Detection Methods

### 3.1. Colloidal Gold Immunochromatographic Assay (GICA)

Colloidal gold is a suspension of gold nanoparticles in water and is known for its optical and molecular recognition properties [70]. Gold nanoparticles have several substantial applications in biomedical research, electron microscopy, and nanotechnology [71,72]. For instance, they can be coated with peptides and glycans for high-sensitivity immunological detection methods that seem to offer great potential for the development of diagnostic assays for specific antibodies in patient sera [73]. The colloidal gold immunochromatographic assay (GICA) uses a cellulose membrane as a carrier and a colloidal gold-labeled antigen or antibody as a tracer [74]. This test, which has been utilized in the diagnosis of influenza A infection, is considered rapid, reliable, and inexpensive [74].

**Figure 4:**
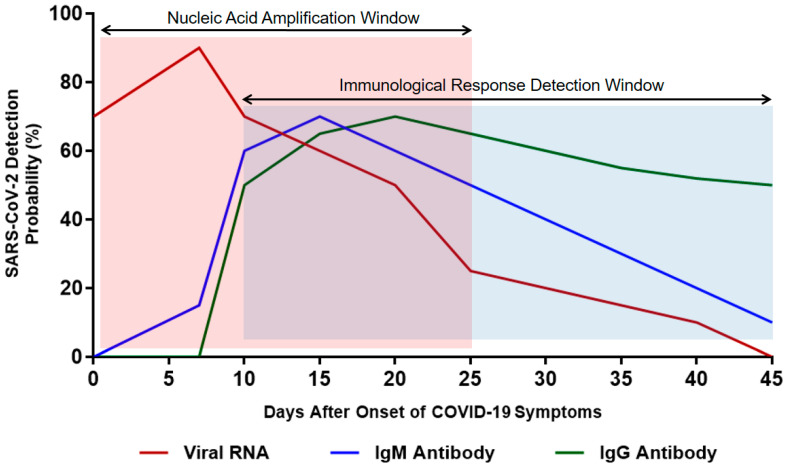
Estimated time interval and detection probability rate of SARS-CoV-2 infection depending on nucleic acid amplification and antibody response. Data were collected from several published reports [75,76,77,78].

Immunoglobulin M (IgM) is the first line of defense against viral infection prior to the production of a long-term immune response provided by immunoglobulin G (IgG) [75]. Therefore, detecting certain antibodies of SARS-CoV-2 in a blood sample serves as a highly sensitive diagnostic technique [76]. Studies have reported that IgM antibodies can be detected following 3 to 6 days of SARS, whereas IgG antibodies are detected after 8 days of infection (Figure 4) [77]. Since SARS-CoV-2 is a member of the same family of viruses that cause SARS (*Coronaviridae*), the presence of IgM and IgG antibodies against them is expected in the blood of COVID-19 patients as an indication of infection [76]. IgM detection is considered an indication of early infection, whereas the presence of IgG antibodies indicates a late viral exposure [78].

Using the colloidal gold immunochromatographic assay (GICA), a simple and fast point-of-care immunoassay was developed that can detect IgM and IgG antibodies against SARS-CoV-2 at the same time [76]. The clinical detection sensitivity and specificity of this test were 88.7% and 90.6%, respectively [76]. Moreover, the test results obtained from peripheral blood samples were consistent with the results of fingerstick blood samples [76]. Therefore, this simple test can be performed for both symptomatic and asymptomatic SARS-CoV-2 carriers at clinics, laboratories, and offsite locations, such as airports or railway stations [76].

One of the limitations of serological tests is their inevitable high analytical error, which could be unpredictable depending on different classes and subclasses of specific antibodies produced during active infection [79]. The presence of endogenous antibodies complicates the detection method through the interaction with biological test reagents [79]. As a result, such tests may end up with congenital inaccuracy, despite implementing the most stringent methodologies and test-specific quality controls [79].

### 3.2. Enzyme-Linked Immunosorbent Assay (ELISA)

The enzyme-linked immunosorbent assay (ELISA) is a regularly used detection assay that utilizes a solid-phase enzyme immunoassay to identify the presence of protein in a liquid sample [80]. This test requires antibodies specific to the ligand to be detected [80]. As a diagnostic tool, ELISA has been implemented in biomedical research, clinical pathology tests, and quality control [81].

Performing an ELISA involves an antibody that has high specificity for capturing a certain antigen [80]. The sample antigens are usually immobilized on a polystyrene microtiter plate, either through surface adsorption or by another antigen-specific antibody (sandwich ELISA) [82]. Following this initial step, an enzyme-linked antibody is added to each well to capture the antigen, and then the unbound antibodies are washed out using a mild buffer [83]. Finally, the addition of a substrate produces a visible signal through hydrolysis, oxidation, or reduction, which can be read at a certain wavelength using a spectrometer to quantify the presence of antigen in each sample [83].

Antibody detection provides crucial clinical data during the course of SARS-CoV-2 infection. The application of ELISA in several studies has provided an empirical value for the regular use of serological testing in the diagnosis and management of COVID-19 patients [84,85,86]. In a study that enrolled 173 SARS-CoV-2 patients, 535 plasma samples were collected during the time of hospitalization and then tested for the dynamic level of total antibodies (Ab), IgM and IgG, which were analyzed according to disease progression [84]. Among those 173 patients, the seroconversion rates for Ab, IgM, and IgG were 93.1%, 82.7%, and 64.7%, respectively [84].

Serological testing has many advantages over PCR due to its high throughput, reduced workload, and rapid detection [86]. However, the antibody response in COVID-19 patients is still not fully investigated since SARS-CoV-2 is a recent virus, and the clinical importance of antibodies is dependent on understanding host antibody response during the period of infection.

### 3.3. Chemiluminescence Immunoassay (CMIA)

Chemiluminescent immunoassay (CMIA) is a biochemical method that is a variation of the standard enzyme immunoassay [87]. This technique has been used as a diagnostic tool in medicine, as well as in various industrial applications. As an advanced serological immunoassay, CMIA is a reliable method for detecting viral infections, including hepatitis C virus (HCV) and Zika virus (ZIKV), due to its wide dynamic range and ultrasensitive luminous intensity [88,89].

The methodological process of CMIA involves enzyme-labeled antibodies to identify unknown biological molecules, such as hormones and proteins [87]. Following an enzymatic reaction, a substrate is converted into a product emitting a photon of light, which can be detected by a luminescent signal instrument [87]. The presence of an antigen is determined by the detection of a particular luminescence [87].

Based on a peptide from the S protein, the CMIA test was developed to detect IgG and IgM against SARS-CoV-2, which was the first assay to identify the antibody response among COVID-19 patients [90]. This test allows a synthetic peptide to be used as an antigen instead of using a whole virus, leading to improved stability and repeatability of this method [90]. Out of 167 sera from patients infected with pathogens other than SARS-CoV-2, none of them showed immunologic reactions to this peptide, which confirms its high specificity [90].

In some cases of pre-existing immune dysfunction and SARS-CoV-2 coinfection, such as with human immunodeficiency virus (HIV) and HCV, a delayed antibody response could affect CMIA detection results up to 42 days postinfection [91]. Therefore, this specific method of detection should be avoided in this group of COVID-19 patients. Further studies are needed to explain the mechanism of this delayed antibody response to SARS-CoV-2 infection among patients with a history of HIV or HCV coinfection.

### 3.4. Fluorescence Labeled Immunochromatographic Assay (FICA)

Fluorescence is the process of light emission by a substance known as fluorophore, which is capable of fluorescing by light [92]. A new incorporated fluorescence immunochromatographic assay (FICA) has shown substantially higher sensitivity and greater dynamic range than color change visual assays [93].

Over the last two decades, fluorescence labeled immunochromatographic assays (FICAs) have become commonly available for the identification of low substance concentrations including toxins and diagnostic biomarkers [94]. They provide numerous promising characteristics, such as high detection sensitivity and various clinical applications in laboratory medicine [94]. The implementation of the FICA principle has enhanced the development of an assay system for the precise quantification of human serum albumin (HSA) using fluorophores, such as Alexa 647 and sulforhodamine B [95].

The principle of FICA was used to develop a SARS-CoV-2 infection diagnostic assay to detect viral nucleocapsid protein in urine samples of COVID-19 patients [96]. In a double-blind clinical trial, NP swabs and urine samples were collected from 239 suspected COVID-19 patients on the same day [96]. The positive results of nucleocapsid protein FICA were 141 out of 208 RT-PCR-confirmed cases, whereas 31 RT-PCR-negative-confirmed cases corresponded directly with their FICA results, indicating 76.4% sensitivity and 100% specificity of this detection method [96]. This accurate and rapid assay provides a simple method for SARS-CoV-2 detection as early as 3 days of infection [96]. In addition, it adds a clinical diagnostic value for the presence of this viral nucleocapsid protein in urine samples, which raises the question of whether SARS-CoV-2 plays a role in inducing renal failure among critically ill COVID-19 patients [96].

**Figure 5:**
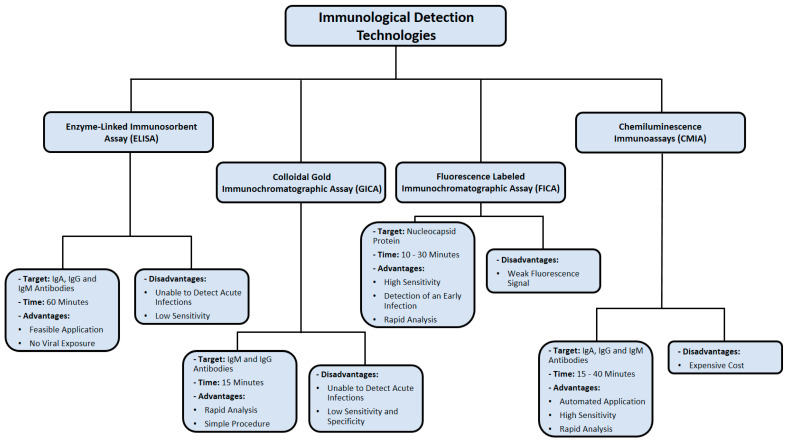
Summary of immunological detection methods used in diagnosing SARS-CoV-2 infection. Data were obtained from several published reports [76,77,78,79,84,85,86,90,91,96,97].

One of the major limitations of FICA is fluorescence quenching, which decreases the emission intensity of a certain fluorophore [97]. This could happen as a result of complex formation, excited state, and energy transfer [97]. Consequently, quenching poses an issue for laser-induced fluorescence in FICA. The immunological detection methods used for SARS-CoV-2 detection are summarized in Figure 5.

## 4. Nucleic Acid Sequencing Methods

### 4.1. Clinical Metagenomic Next-Generation Sequencing (mNGS)

Metagenomic sequencing is characterized by a comprehensive analysis of all nucleic acids in one clinical sample, which may include host and microbial genetic material (DNA or RNA). Therefore, mNGS identifies infectious microorganisms without prior knowledge of what specific pathogen is being detected [98]. This makes mNGS a powerful diagnostic tool, especially when other more direct methods, such as PCR, are unable to determine a certain infection [99]. This method has been applied to various sample types, including blood, cerebrospinal fluid, respiratory samples, and gastrointestinal fluid [100].

A typical mNGS workflow consists of clinical sample acquisition, followed by RNA or DNA extraction [100]. Then, high-throughput sequencing is performed, in which nucleic acid fragments of the library are sequenced according to a selected platform [96]. Several factors play a major role in choosing a sequencing platform, such as personal experience and laboratory objectives [100]. The Illumina MiSeq is the most commonly used platform for infectious disease diagnostics and pathogen discovery for public health reasons [101]. Once sequencing is obtained, data interpretation and bioinformatics analysis require appropriate computational resources to identify each specific pathogen.

The use of the mNGS approach for the identification of SARS-CoV-2 was successfully achieved on RNA extracted from the BALF of two patients experiencing unusual severe pneumonia in Wuhan, China, on 2 January 2020 [102]. This method rapidly identified the newly emerging virus, as it was the only pathogen in the sample with a relatively high abundance level (1.5% and 0.62% of total RNA sequenced) [102]. Additionally, five BALF samples of patients experiencing similar symptoms of acute respiratory distress syndrome in the same area were analyzed by mNGS [103]. Data revealed the presence of SARS-CoV-2 in all five patients with 99.8% nucleotide identities among viral isolates [103]. These isolates also showed 79% nucleotide identity with the sequence of SARS-CoV (GenBank NC_004718) and 51.8% identity with the sequence of MERS-CoV (GenBank NC_019843) [103].

The ability to detect SARS-CoV-2 infection by mNGS was also applied on minimally invasive patient samples collected through nasopharyngeal (NP) swabs [104]. By using the direct Oxford Nanopore third-generation (long read) metatranscriptomic and metagenomic sequencing, 50 NP patient samples were analyzed to detect SARS-CoV-2 infection [104]. In addition to confirming the presence of SARS-CoV-2 in NP swab samples, using mNGS demonstrated that this newly emerging virus causes a significant shift in the respiratory microbiome [104]. Consequently, the application of mNGS can be used as a method for diagnosing SARS-CoV-2 coinfections without the need for amplifying a viral target [104].

Despite the successful advancement of mNGS applications, a key limitation of its clinical use is its low sensitivity against the background microbiome, which complicates the process and makes it unclear whether the detected microorganism is a colonizer or a pathogen [105]. Moreover, the universal standards for test validation, reproducibility, and quality assurance for clinical mNGS assays are lacking [105]. SARS-CoV-2 cDNA is very difficult to detect during the incubation period [16]. As a result, researchers have optimized mNGS by incorporating amplicon-detecting radiolabeled probes into existing protocols [16]. Despite its enhanced detection sensitivity, this approach requires cumbersome downstream processing, which is ineffectual in a routine laboratory setting [16]. The major obstacles of implementing mNGS in patient care settings are characterized by its expensive cost, clinical utility, and regulatory considerations [98]. To date, clinical mNGS outcome data mostly consist of case reports, which provide a glimpse into the future application of mNGS in public health settings [98].

### 4.2. Nanopore Third-Generation Sequencing (NTS)

Nanopore sequencing (NTS) is a third-generation sequencing method that involves the sequencing of polynucleotides from DNA or RNA without chemical labeling or PCR amplification of the tested samples [106]. This method offers relatively quick sample processing and high testing mobility [107]. As a result, it has been applied in identifying many viruses such as the Ebola virus, haplotyping, and monitoring antibiotic resistance [108]. Biological NTS uses transmembrane proteins known as porins, which are distributed across the surface of lipid membranes, creating a low translocation velocity to facilitate nucleic acid movement [109]. In contrast, the solid-state NTS method utilizes porous metal alloy substrates that allow nucleic acid to pass through [110].

Using SARS-CoV-2-infected Huh7 cells, the longest (26 kb) contiguous read was mapped to a viral reference genome [111]. In addition, this approach, which bypasses reverse transcription and amplification of RNA, detected methylation sites in viral RNA [111]. The detection specificity of NTS for SARS-CoV-2 was 100%, and parallel testing with RT-PCR kits showed that NTS identifies more positive samples [112]. The use of NTS also effectively monitors mutation in RNA sequences, classifies subtypes of SARS-CoV-2, and detects other respiratory viruses in the same sample. Therefore, NTS is considered an appropriate test for SARS-CoV-2 detection, and this method may be further expanded to identify more pathogens [112,113]. The application of NTS assisted in identifying the nonstructural protein 1 (nsp1) gene, which is located at the 5′ end of the SARS-CoV-2 genome, and was highly expressed in NP swab samples of COVID-19 patients who presented with various clinical severity symptoms [114]. These findings resulted in the development of a novel nsp1 RT-PCR assay with highly specific primers to SARS-CoV-2 [114].

**Figure 6:**
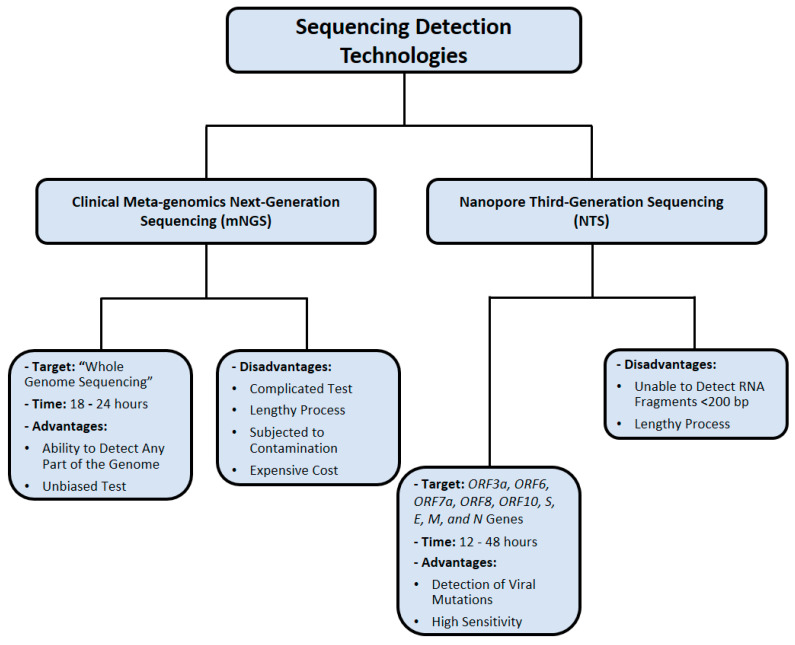
Nucleic acid sequencing methods used for SARS-CoV-2 detection. Data were obtained from several published reports [102,103,104,105,111,112,113,114,115].

A few challenges of NTS implementation are characterized by the requirement of technical bioinformatics expertise, high cost, and lengthy time [115]. However, Oxford Nanopore Technologies (ONT) is the recently developed technological innovation NTS, which addresses these challenges by providing a user-friendly platform that saves time, but this method is still limited by the issue of base-calling accuracy in comparison with other platforms [115]. Figure 6 summarizes the nucleic acid sequencing methods that were utilized in SARS-CoV-2 detection.

## 5. CRISPR-Based Detection Methods for SARS-CoV-2 Infection

The CRISPR (clustered regularly interspaced short palindromic repeats) locus was first observed in 1980 in *Escherichia coli* and has since been noticed in 45% of bacterial genomes and 84% of archaeal genomes [116]. It was not until 2007 that the function of the CRISPR locus was identified in *Streptococcus thermophilus* after the integration of bacteriophage fragments into the locus resulted in resistance against the virus [116]. In bacteria and archaea, the CRISPR locus, along with cas (CRISPR-associated) genes, provides an adaptive immune system against viruses, plasmids, and other foreign nucleic acids [116].

The CRISPR–Cas system has mostly been used as a “molecular scissor” for genome editing since the discovery of its RNA-programmable site-specific DNA cleavage in 2012 [117]. However, a recent area of development is using the CRISPR–Cas system for nucleic acid detection for point-of-care molecular diagnostics due to its high sensitivity, specificity, and reliability [118]. The SHERLOCK (specific high-sensitivity enzymatic reporter unlocking) and DETECTR (DNA endonuclease-targeted CRISPR trans reporter) systems were developed through the combination of recombinase polymerase amplification (RPA)—an isothermal amplification method—with Cas13 and Cas12 nucleases, which indiscriminately cleave nontarget single-stranded nucleic acids surrounding the CRISPR RNA–target duplex (termed “collateral cleavage”) [118]. Probes with a fluorophore and quencher are added to the reaction so that upon binding of the CRISPR–Cas system to its target, Cas13 or Cas12 will cleave nearby probes, releasing the fluorophore from its quencher and allowing for the production of a fluorescent signal [118].

The lateral flow assay SARS-CoV-2 DETECTR is a CRISPR–Cas12-based assay developed to detect SARS-CoV-2 in approximately 30 min [119]. For this assay, RNA is extracted from nasopharyngeal or oropharyngeal swabs and is simultaneously reverse-transcribed and isothermally amplified for the E and N genes of SARS-CoV-2 using loop-mediated isothermal amplification (RT-LAMP) [119]. CRISPR–Cas12-based detection is then used wherein the cleavage of a reporter molecule confirms viral detection [119]. Upon analyzing PCR-positive COVID-19 patient respiratory samples and nasopharyngeal swabs of patients with influenza and common human seasonal coronavirus and healthy donors, SARS-CoV-2 DETECTR showed 90% sensitivity and 100% specificity for the detection of SARS-CoV-2, demonstrating a performance analogous to the CDC RT-qPCR Diagnostic Panel [119]. Additionally, when using synthetic in vitro transcribed RNA gene targets, SARS-CoV-2 DETECTR did not show any cross-reactivity and successfully distinguished SARS-CoV-2 from bat SARS-like coronavirus (bat-SL-CoVZC45) and SARS-CoV [119].

In August 2020, a similar CRISPR-based SARS-CoV-2 detection assay, CRISPR-COVID, was developed using the nuclease Cas13a, which also possesses unique collateral cleavage activity [120]. This 40 min method targets the *Orf1ab* gene of SARS-CoV-2 by RPA and CRISPR–Cas13a and then cleaves nearby probes, allowing fluorescence for a positive test result [120]. CRISPR-COVID had a consistent limit of detection of 7.5 copies, but 6 out of 10 replicates detected 2.5 copies, and 2 out of 10 replicates detected 1.25 copies, indicating a sensitivity of nearly a single copy [120]. The assay showed great specificity as seen by the lack of false positives when tested in a panel of microbes that included bacterial respiratory infections, other human coronaviruses, and viral respiratory infections [120]. Furthermore, out of 52 mNGS-confirmed COVID-19 cases, 100% were detected via CRISPR-COVID, and among 62 negative cases, there were no false-positive results [120].

The all-in-one dual CRISPR–Cas12a (AIOD-CRISPR) assay was developed for real-time or visual detection of SARS-CoV-2 using two CRISPR–Cas12a complexes with different CRISPR RNA sequences to target two distinct sites within the N gene of SARS-CoV-2 [118]. In a single reaction solution, the target sequence, CRISPR–Cas12a complexes, RPA primers, single-stranded DNA fluorophore-quencher reporter, strand-displacement DNA polymerase, single-stranded DNA binding protein, and recombinase are combined and incubated at 37 °C for 40 min, eliminating the separate amplification step and transfer of the amplified product [118]. When testing the detection specificity of AIOD-CRISPR, the assay consistently detected down to approximately five copies of RNA targets in both real-time and visual detection without any cross-reactivity with SARS-CoV or MERS-CoV [118]. Clinical swab samples of COVID-19-positive and negative individuals were also used to validate the assay [118]. AIOD-CRISPR successfully detected SARS-CoV-2 in all COVID-19-positive samples and produced results consistent with the CDC’s RT-qPCR assay [118].

Although CRISPR-based detection is a relatively low-cost procedure to carry out, multistep procedures with separate amplification and detection steps, such as DETECTR, can potentially be complicated and have a greater risk of carry-over contamination [118]. However, due to its highly sensitive and specific nucleic acid detection capabilities, use of widely accessible protocols and reagents, and much quicker turnaround time, the application of the CRISPR–Cas system for diagnostic purposes is highly favorable [118,119,120].

Additionally, CRISPR–Cas detection provides a financially competitive diagnostic with similar specificity to NGS-based assays [113,114,115]. CRISPR-based detection mechanisms also circumvent the need for lab equipment such as thermocyclers in PCR-based detection mechanisms, allowing for wider accessibility and rapid use as a point-of-care diagnostic [118,119,120].

## 6. Direct Isolation of SARS-CoV-2 from Clinical Samples of COVID-19 Patients

Viral shedding has been reported in SARS-CoV patients, which was detected in respiratory and stool samples for up to 7 and 18 weeks, respectively [121]. In contrast, viral shedding in MERS-CoV patients has not been reported frequently, and it could not be isolated from the stool or serum samples [122]. Although studies have suggested that SARS-CoV-2 RNA is not detectable in the upper respiratory tract following 21 days of infection, viral shedding from respiratory specimens was observed for up 37 days [123]. While SARS-CoV-2 RNA was detected in serum, urine, and stool samples of COVID-19 patients several days postinfection, the virus could not be isolated, which suggests a low risk of transmission via stool or urine [124]. However, the presence of gastrointestinal symptoms in hospitalized COVID-19 patients suggests a potential role of SARS-CoV-2 in gastrointestinal manifestations and a possibility of fecal–oral transmission as reported by the American Gastroenterological Association [125]. The direct isolation of SARS-CoV-2 is considered useful for studying drug susceptibility, and the transmembrane serine protease 2 (TMPRSS2)-expressing VeroE6 cell line enhances the useful isolation and propagation of SARS-CoV-2 [126].

## 7. Concluding Remarks

The ongoing COVID-19 pandemic caused by SARS-CoV-2 infection continues to spread throughout the whole world. In January 2020, WHO declared this outbreak a public health emergency of international concern. As of March 2021, over 110 million cases of COVID-19 have been confirmed, with more than 2.5 million fatalities, which makes it one of the deadliest pandemics in history. The clinical manifestations of COVID-19 are very similar to those of several respiratory illnesses, thus making it almost impossible to detect by using clinical imaging techniques such as CT without performing additional ultrasensitive molecular diagnostic tests to confirm or rule out COVID-19 diagnosis. Therefore, developing detection assays for SARS-CoV-2 infection and a clear understanding of test result interpretations is of paramount importance.

Nucleic acid amplification technologies (RT-qPCR, dPCR, mPCR, and LAMP) are considered the gold standards for detecting SARS-CoV-2 infection since these methods are fast, highly sensitive, and relatively cost-effective. However, there are several concerning issues facing most nucleic-acid-based detection methods, including false-positive or false-negative probability and the requirement of expensive thermal cycler equipment. Immunological methods carry some advantages over nucleic-acid-based techniques as they provide information regarding the presence of serum antibodies against SARS-CoV-2, which can be detected beyond 4 weeks of infection. However, antibodies are not produced until 5 days after the onset of symptoms, which makes serological assays only complementary confirmations to PCR assays during early phases of infection (IgM antibodies) and an indication of late exposure after disease remission (IgG antibodies). Health care providers rely on PCR testing especially when a person has COVID-19 symptoms, while antigen testing is most appropriate for surveillance or screening, such as when colleges are trying to determine the prevalence of the virus on campus. Combining both antigen and PCR tests will not provide a clinical value for COVID-19 patients since the endpoint result will be determined through PCR testing.

The application of sequencing technologies (mNGS and NTS) for SARS-CoV-2 detection provides several advantages over PCR-based methods, such as detection of coinfections without the need for amplifying a viral gene and identifying novel genes to be used as PCR test amplification targets. Despite these advancements, the sequencing methods lack universal validation standards, and they are relatively expensive in comparison with other available methods. To date, clinical mNGS data mostly consist of case reports, providing a glimpse into the future application of mNGS in public health settings. Oxford Nanopore Technologies (ONT) is the recent technological NTS innovation, which addresses major NTS challenges by providing a fast user-friendly platform. As mentioned in Table 1, we summarized the pros and cons of SARS-CoV-2 detection technologies. In conclusion, SARS-CoV-2 clinical diagnostics and detection technologies play a major role in controlling the COVID-19 outbreak by enabling health care professionals to direct resources and efforts to patients to ultimately curb the spread of infections and reduce viral mortality.

## Figures and Tables

**Table 1:** Summary of the main advantages and disadvantages of methods used to detect SARS-CoV-2 infection.

Technique	Advantages	Disadvantages	References
Nucleic Acid Amplification Techniques			
RT-qPCR	High sensitivity and accuracy, absolute and relative quantification, low risk of contamination	Risk of false-positive or negative detection, primers, and reaction efficiency can affect testing outcome	[14,21,22]
dPCR	High sensitivity and accuracy, absolute detection	Expensive	[34,35,38,40,41]
Multiplex PCR	High sensitivity and accuracy, ability to detect multiple targets, cost-effective, simple workflow	Further optimization required for primer and reaction conditions, potential for contamination, potential for reaction failure	[43,54,55,59,60]
LAMP	High sensitivity, thermal cycler not required, extensive sample processing not needed, quick, cost-effective	Sensitivity depends on sample complexity, difficult to prepare appropriate primer designs, lacks clinical data	[62,64,65,69]
Immunological Detection Methods			
GICA	Simple workflow, rapid analysis, cost-effective	Cannot detect acute infections, low sensitivity and specificity	[74,79]
ELISA	Simple workflow, rapid detection, no viral exposure	Cannot detect acute infections, low sensitivity	[86]
CMIA	High sensitivity, automated application, rapid analysis	Expensive, results may not be accurate in the context of pre-existing immune dysfunction	[90,91]
FICA	High sensitivity, can detect early infection, rapid analysis	Fluorescence quenching	[93,94,96,97]
Nucleic Acid Sequencing Methods			
mNGS	Can detect any part of the genome, unbiased	Complicated and lengthy process, prone to contamination, expensive	[98,105]
NTS	High sensitivity, can detect viral mutations, quick sample processing	Lengthy process, unable to detect RNA fragments < 200 bp, expensive	[107,112,113,114,115]
CRISPR-Based Detection Methods			
CRISPR	Ultrasensitive, high specificity, rapid analysis	Multistep process is prone to contamination	[118,119,120]

## Data Availability

Not applicable.

## Abbreviations

Ab	total antibodies
ACE2	angiotensin-converting enzyme 2
AIOD-CRISPR	all-in-one dual CRISPR–Cas12a
BALF	bronchoalveolar lavage
β-CoV	betacoronavirus
bat-SL-CoVZC45	bat SARS-like coronavirus
CDC	Center for Disease Control
CMIA	chemiluminescent immunoassay
COVID-19	coronavirus disease 2019
CRISPR	clustered regularly interspaced short palindromic repeats
Ct	Relative threshold
CT	computed tomography
ddPCR	droplet digital PCR
DETECTR	DNA endonuclease-targeted CRISPR trans reporter
dPCR	digital polymerase chain reaction
E	envelope protein
ELISA	enzyme-linked immunosorbent assay
FDA	Food and Drug Administration
FICAs	fluorescence labeled immunochromatographic assays
GICA	colloidal gold immunochromatographic assay
HAS	human serum albumin
HCV	hepatitis C virus
HIV	human immunodeficiency virus
IgG	immunoglobulin G
IgM	immunoglobulin M
LAMP	loop-mediated isothermal amplification
LoD	limit of detection
M	matrix protein
MERS-CoV	Middle East respiratory syndrome coronavirus
MHV	murine coronavirus
mNGS	clinical metagenomic next-generation sequencing
mPCR	multiplex PCR
N	nucleocapsid protein
NP	nasopharyngeal
NPV	negative predictive value
nsp	nonstructural protein
NTS	nanopore sequencing (NTS)
ONT	Oxford Nanopore Technologies
PCR	polymerase chain reaction
RdRP	RNA-dependent RNA polymerase
RPA	recombinase polymerase amplification
RTFQ-PCR	real-time fluorescent quantitative PCR
S	surface glycoprotein
SARS-CoV	severe acute respiratory syndrome virus
SARS-CoV-2	severe acute respiratory syndrome coronavirus 2
SHERLOCK	specific high-sensitivity enzymatic reporter unlocking
TMPRSS2	transmembrane serine protease 2
WGS	wide-genome sequencing
WHO	World Health Organization
ZIKV	Zika virus
